# A novel 3D spheroid model of rheumatoid arthritis synovial tissue incorporating fibroblasts, endothelial cells, and macrophages

**DOI:** 10.3389/fimmu.2023.1188835

**Published:** 2023-07-20

**Authors:** Eva M. L. Philippon, Lisanne J. E. van Rooijen, Fatemeh Khodadust, Jan Piet van Hamburg, Conny J. van der Laken, Sander W. Tas

**Affiliations:** ^1^ Department of Rheumatology & Clinical Immunology, Amsterdam Rheumatology & Immunology Center, Amsterdam University Medical Centers, University of Amsterdam, Amsterdam, Netherlands; ^2^ Department of Experimental Immunology, Amsterdam University Medical Centers, University of Amsterdam, Amsterdam, Netherlands

**Keywords:** rheumatoid arthritis, 3D model, endothelial cells, fibroblasts, macrophages

## Abstract

**Objective:**

Rheumatoid Arthritis (RA) is a progressive and systemic autoimmune disorder associated with chronic and destructive joint inflammation. The hallmarks of joint synovial inflammation are cellular proliferation, extensive neoangiogenesis and infiltration of immune cells, including macrophages. *In vitro* approaches simulating RA synovial tissue are crucial in preclinical and translational research to evaluate novel diagnostic and/or therapeutic markers. Two-dimensional (2D) settings present very limited *in vivo* physiological proximity as they cannot recapitulate cell-cell and cell-matrix interactions occurring in the three-dimensional (3D) tissue compartment. Here, we present the engineering of a spheroid-based model of RA synovial tissue which mimics 3D interactions between cells and pro-inflammatory mediators present in the inflamed synovium.

**Methods:**

Spheroids were generated by culturing RA fibroblast-like-synoviocytes (RAFLS), human umbilical vein endothelial cells (ECs) and monocyte-derived macrophages in a collagen-based 3D scaffold. The spheroids were cultured in the presence or absence of vascular endothelial growth factor (VEGF) and fibroblast growth factor 2 (bFGF) or RA synovial fluid (SF). Spheroid expansion and cell migration were quantified for all conditions using confocal microscopy and digital image analysis.

**Results:**

A novel approach using machine learning was developed to quantify spheroid outgrowth and used to reexamine the existing spheroid-based model of RA synovial angiogenesis consisting of ECs and RAFLS. A 2-fold increase in the spheroid outgrowth ratio was demonstrated upon VEGF/bFGF stimulation (*p*<0.05). The addition of macrophages within the spheroid structure (3.75x10^4^ RAFLS, 7.5x10^4^ ECs and 3.0x10^4^ macrophages) resulted in good incorporation of the new cell type. The addition of VEGF/bFGF significantly induced spheroid outgrowth (*p*<0.05) in the new system. SF stimulation enhanced containment of macrophages within the spheroids.

**Conclusion:**

We present a novel spheroid based model consisting of RAFLS, ECs and macrophages that reflects the RA synovial tissue microenvironment. This model may be used to dissect the role of specific cell types in inflammatory responses in RA, to study specific signaling pathways involved in the disease pathogenesis and examine the effects of novel diagnostic (molecular imaging) and therapeutic compounds, including small molecule inhibitors and biologics.

## 1 Introduction

Rheumatoid arthritis (RA) is a chronic autoimmune joint disease affecting 0.5-1.0% of the population worldwide. RA manifests with destructive synovial inflammation causing joint tenderness, pain and swelling. If ineffectively treated, RA can lead to severe cartilage degradation, bone erosion and irreversible disability (1–3). Despite major advancements in managing RA symptoms with disease-modifying antirheumatic drugs (DMARDs), around 20-40% of the patients still experience treatment failure, including limited efficacy and (secondary) nonresponse, which may increase disability over time due to inadequate treatment (4). The diversity of the cellular component in the RA synovium was thereby dissected and distinct histomorphological patterns were identified according to the composition, organization and localization of cellular infiltrates. Three main synovial “pathotypes” were recognized, namely the fibroblastic pauci-immune pathotype, the macrophage-rich diffuse-myeloid pathotype, and the lympho-myeloid pathotype characterized by prominent lymphocyte infiltration (5–11). The pathotypes have been described to correlate with disease activity and/or severity, as well as response to therapy. These findings underlie the importance of the cellular composition of the RA synovium and the interaction between cell types in establishing novel RA treatment strategies.

The RA synovial tissue is characterized by a hyperplastic intimal lining layer of fibroblast-like synoviocytes (FLS) and macrophage-like synoviocytes (MLS) which interact with a variety of activated immune cells multiplying in the sublining layer. The complex crosstalk among the numerous cell types present in the RA synovium leads to the production of a wide array of cytokines and pro-inflammatory molecules responsible for the chronic inflammation (12–17). Pro-angiogenic factors activate endothelial cell proliferation and upregulation of adhesion molecules as part of the angiogenesis process (18). Extensive blood vessel formation allows immune cells to massively infiltrate the synovial tissue. Among them, pro-inflammatory macrophages further contribute to the excessive accumulation of cytokines and chemokines at the site of inflammation (12–17). Moreover, RAFLS exhibit an invasive and aggressive phenotype, capable of invading the local synovial tissue environment. They harbor the ability of remodeling the extracellular matrix (ECM) through the secretion of matrix-degrading enzymes and the expression of increased levels of adhesion molecules (18–23). Behaving like invasive tumor, this highly vascularized expanding synovial tissue mainly composed of invasive FLS and macrophages, known as “pannus”, causes disruption of joint cartilage and bone erosion (5, 12, 13, 19–22).

Many *in vitro* systems modelling the RA synovial tissue have been developed, which are used for drug testing, mechanistic research and clinical translation (24, 25). Diverse strategies are currently being explored to find the right balance between experimental feasibility, reproducibility, and *in vivo* physiological proximity. While 2D settings fail at simulating the physiological *in vivo* situation, 3D culture approaches facilitate cell-cell and cell-matrix interactions involved in key cellular processes such as cell proliferation, migration and differentiation. Due to their pivotal role in synovial inflammation, RAFLS are found in most of the 3D models of synovial tissue (24, 25). Cultured as micromasses (26, 27) or combined with peripheral CD14^+^ monocytes (28, 29), these models reflected key features of the RA synovium after TNF exposure, including the formation a lining layer at the outer surface, the alteration of macrophage phenotype and the expression of pro-inflammatory cytokines. Additionally, neovascularization and neoangiogenesis have also been described in RA models with the co-cultures of RAFLS and mesenchymal stromal cells or vascular ECs (30, 31). Similarly, we previously established a spheroid-based model of synovial angiogenesis incorporating RAFLS and ECs (32). The spheroids were stimulated with the growth factors VEGF/bFGF, key regulators of angiogenesis, or cultured in the presence of RA synovial fluid (SF), known to contain pro-angiogenic factors (33). In the current study, we incorporated monocyte-derived macrophages to our existing model to further improve its ability to mimic the *in vivo* RA synovial tissue and reflect the interactions of RAFLS and ECs with immune cells in pathological processes of RA, which includes synovial inflammation, angiogenesis and pannus formation.

Of note, diverse quantitative approaches have been developed to quantify cell migration in 3D models, particularly to describe angiogenesis. The ImageJ plugin “Sprout morphology” and the Matlab-based algorithm “AQuTAS” were both created to assess angiogenic sprouting from EC spheroids (34, 35). Comparably to EC sprouting, FLS have previously been documented to extend and retract a so-called dendritic network of extensions in collagen matrices (36). Moreover, RAFLS and ECs showed coordinated migration *in vitro*, remarkably overlapping and providing mutual structural support during EC sprout formation (32, 37). To capture the migratory capacities of the multiple cell types involved in inflammation and disease progression in the RA synovial tissue, we developed a new quantitative approach measuring the spheroid outgrowth area covered by the cells altogether migrating throughout the matrix.

Here, we present a new tool to quantify spheroid expansion and the development of a novel spheroid-based 3D model of RA synovial tissue incorporating RAFLS, ECs, and monocyte-derived macrophages. This innovative model aims to mimic the interactions between these cell types and enables the investigation of their role in RA synovial inflammation.

## 2 Methods

### 2.1 Patient materials

FLS were isolated from RA patients as previously described (38). A synovial fluid cocktail was made by pooling synovial fluid of 11 RA patients. ECs were collected from human umbilical vein from healthy donors and monocytes were isolated from fresh blood or buffy coat (Sanquin, Amsterdam, NL) from healthy donors. Patient consent was obtained by all participants in written format according to the Declaration of Helsinki and the study was approved by the medical ethics committee of the Academic Medical Centre, University of Amsterdam, Amsterdam, the Netherlands.

### 2.2 Cell culture

RAFLS were cultured in DMEM (Gibco, Carlsbad, CA, USA) supplemented with 10% fetal calf serum (FCS) (Biowest, Ennigerloh, Germany), 100 μg/ml penicillin–streptomycin (Gibco), 2 mM L-glutamine (Gibco), 10 mM HEPES (Gibco) and 250 μg/ml gentamicin (Gibco) up to passage 8. ECs were expanded up to passage 4 on 1% gelatin in M199 (Gibco) containing 20% FCS, 100 μg/ml penicillin–streptomycin, 50 μg/ml heparin, 12.5 μg/ml endothelial cell growth supplement (ECGS) (Corning, NY, USA) and 2 mM L-glutamine. Monocytes were seeded at 3x10^5^ cells/well and differentiated into “pro-inflammatory” macrophages in IMDM (Gibco) complemented with 5% FCS, 250 μg/ml gentamicin and 20 ng/ml GM-CSF (Gibco) for 6-7 days including the renewal of medium at day 3.

### 2.3 Spheroid co-culture assay

**Figure 1:**
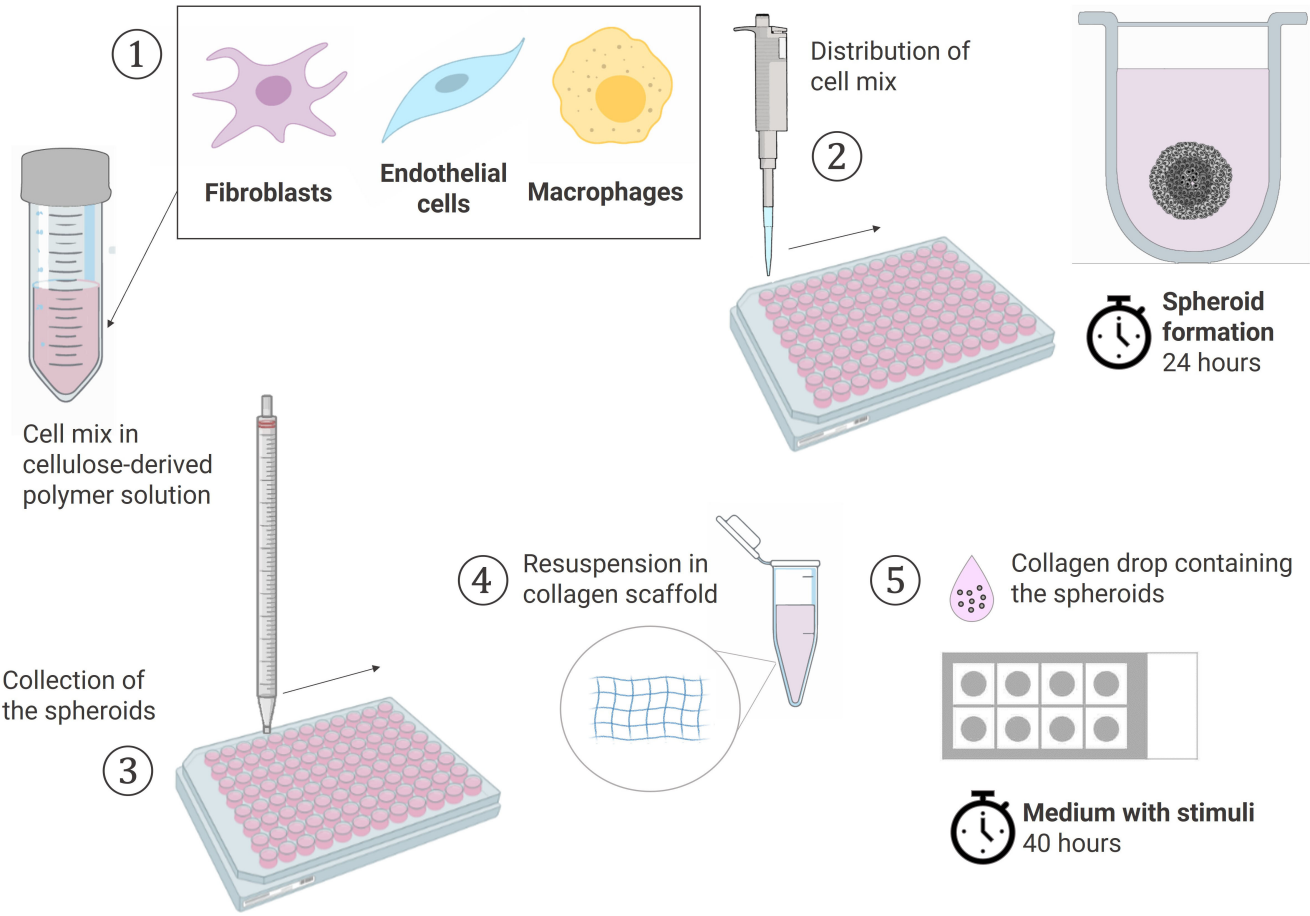
Schematic workflow of the spheroid formation and the sprouting assay in a collagen-based 3D scaffold. (1) Cells pooled together in 20% Methocell solution (2) Spheroid formation: distribution of 150 µl cell mix/well into a 96 U-well suspension plate and incubation at 37°C for 24h (3) Collection of the spheroids with a 10 ml pipet (4) Spheroids resuspended in a 1.5 mg/ml collagen solution (5) Sprouting assay: 20 µl of collagen solution containing the spheroids placed dropwise using the sandwich method in a 8-chamber slide and cultured in the suitable media containing stimuli at 37°C for 40h. Created with BioRender.com.

The experiments were performed with different combinations of donors of RAFLS, ECs and macrophages each time. RAFLS, ECs, and macrophages were respectively pre-incubated in 2 µM CellTracker™ Orange CMTMR, Red CMTPX and Green CMFDA dyes (Invitrogen) in culture medium. The cells were harvested, counted and pooled together in 15 ml of 20% methocel solution (v/v) in EGM-2 Endothelial Cell Growth Medium-2 (Lonza, Basel, Switzerland) (**Figure 1**). Methocel solution was prepared by dissolving 6 g methylcellulose in 500 ml M199 medium. The cell mix containing 3.75x10^4^ RAFLS, 7.5x10^4^ ECs and the suitable number of macrophages was distributed into a 96 U-well suspension plate (Greiner BioOne, Stonehouse, UK) and spheroids were formed overnight at 37°C. Next, a 200 µl solution of 1.5 mg/ml of rat-tail collagen type-I (BD Biosciences, Oxford, UK) was prepared in EGM-2 medium and the pH was neutralized by 2 µl NaOH (1M). A collagen drop (20 µl) was distributed in each well of an 8-well Ibitreat chamber slide (Ibidi, Martinsried, Germany) and set at 37°C for 30 min for complete polymerization. Spheroids were collected from the plate into a 15 ml tube, washed twice with PBS and resuspended in an equivalent 200 µl collagen solution. The same droplet volume was deposited over the first layer in each well to sandwich the spheroids between the two collagen drops and control the distance at which the spheroids were situated above the glass bottom of the well. The 3D collagen-based scaffold was set at 37°C for 1 hour. EC starvation medium was prepared as previously described with only 2% FCS. The medium was left with no stimulant or was supplemented with either 10 ng/ml VEGF/bFGF or 20% SF. The spheroids were incubated in 400 µl/well of the suitable media for 40 h. Culture supernatants were collected and placed at -20°C for further analysis.

### 2.4 ELISA

TNF (Human TNF alpha Monoclonal antibody pair (MAb11), eBioscience™) and IL-6 (Human IL-6 antibody pair, U-CyTech) were quantified in the collected spheroid culture supernatants using commercially-available ELISA according to manufacturer’s instructions.

### 2.5 RNA isolation and quantitative RT-PCR

The collagen drops containing the spheroids were washed twice with equivalent volume of RPMI 1640 Medium (Gibco). The collagen gels were removed from the wells with a P1000 pipet and placed in a 1.5 ml tube containing 50 µg/ml Liberase™ TM Research Grade solution in RPMI (Sigma-Aldrich, St. Louis, MO, USA). At least 20 collagen drops were pooled together per condition to have enough cells for RNA isolation. The collagen matrix was digested by incubating in Liberase solution at 37°C, shaking at 1000 rpm for 10 min. The spheroids were shortly span down at max speed and washed twice with PBS. The spheroids were dissociated into a single-cell suspension by incubating in Accutase^®^ (STEMCELL Technologies, Vancouver, Canada) at 37°C, shaking at 1400 rpm for 15 min. The cells were span down at 1700 rpm for 5 min and washed twice in PBS to proceed with RNA isolation. RNA was isolated using GenElute™ Mammalian Total RNA Miniprep (Sigma-Aldrich) according to manufacturer’s instructions. cDNA was synthesized using ThermoFisher Scientific first strand cDNA synthesis kit (Thermo Scientific, Waltham, MA, USA). Quantitative RT-PCR was done by Fast SYBR™ Green Master Mix (Applied Biosystems, Waltham, MA, USA) using appropriate primers (primer sequences available upon request). Analysis was done using the QuantStudio™ 3 Real-Time PCR System (Applied Biosystems).

### 2.6 Spheroid imaging and quantification

For imaging the spheroids, the collagen drops were washed twice with equivalent volume of Hanks’ Balanced Salt Solution (HBSS) (Gibco). The spheroids embedded in the collagen matrix were fixed by adding 400µl/well of 4% paraformaldehyde (w/v) in HBSS and letting the slides at room temperature (RT) for 1h. The fixed spheroids were washed twice with equivalent volume of phosphate-buffered saline (PBS) and stored in PBS at 4°C in the dark until imaging with a Leica TCS SP8-X confocal microscope (10X) (Leica Camera AG, Wetzlar, Germany). CI convert was used to compile the confocal picture Z-stacks in 2D projection while preserving the distinct pixel values for all imaging channels. The converted pictures were imported to QuPath image analysis software and the suitable channels were used for subsequent quantitative measurement (detailed protocol in **Supplementary Material 1**). The region covered by the cells that migrated throughout the matrix, comprising both associated and non-associated cells, was segmented by pixel classification and discriminated from the region occupied by the core. Areas were calculated for each picture by applying the most suitable pixel classifier previously trained on a pool of representative pictures. Output analysis was checked for each picture and readjusted with a different trained pixel classifier if needed. QuPath analysis tools such as “Cell Detection” and “Calculate Features” were also used to detect and count the cells outside of the core and add intensity values of the different regions respectively. Subsequently, the ratio of the spheroid outgrowth to the core area was calculated, as well as the integrated density of the specific stained cells ( Specific Mean Intensity×Area ) in the respective areas and their percentage of the total integrated density. Measurement parameters were quantified for 2-7 isolated spheroids (biological replicates) and the averages were calculated for each condition.

### 2.7 Immunohistochemistry and immunofluorescence

Following fixation, the collagen gels containing the spheroids were incubated in 15% sucrose solution in PBS for 1 hour at RT (400µl/well). This step was repeated with 30% sucrose solution. For subsequent immunofluorescence imaging, the collagen gels were transferred to cassettes with a thin spatula, covered by Tissue-Tek O.C.T. Compound (Sakura Finetek, California, USA) and snap-frozen in liquid nitrogen. The frozen samples were stored at -80°C until cryosectioning (5 µm) using a cryostat. The cryosections were dried overnight at RT, mounted on microscope slides and directly imaged with a Leica TCS SP8-X confocal microscope (20X). For subsequent hematoxylin and eosin (HE) staining, liquid 1% agarose gel was added to the collagen gels containing the spheroids (400µl/well) and set at RT until full polymerization. The agarose blocks were scooped out from each well with a thin spatula, placed in cassettes and stored in 70% ethanol until paraffin embedding. A sliding microtome was used to cut 5 µm sections from the paraffin embedded samples, followed by deparaffination and HE staining (39).

### 2.8 Statistical analysis

Statistical analyses were performed using GraphPad Prism v8.2.1 (Graphpad Software Inc.). A *p*<0.05 was considered statistically significant. Differences were analyzed by parametric or non-parametric one-way analysis of variance with repeated measures (RM one-way ANOVA) followed by multiple comparisons test or a ratio paired t-test when indicated.

## 3 Results

### 3.1 Application of a machine learning quantitative analysis (QuPath) to measure spheroid outgrowth and morphological changes

**Figure 2:**
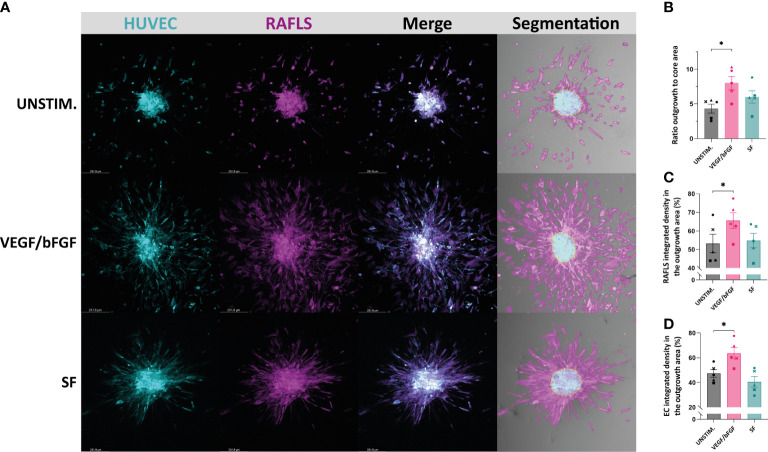
Application of a machine learning quantitative analysis (QuPath) in the pre-established spheroid-based model of RA synovial angiogenesis to measure spheroid outgrowth and morphological changes. Spheroids were either left unstimulated (UNSTIM.), stimulated with VEGF/bFGF (10 ng/ml) or SF (20%). Representative confocal Z-stack projection pictures (10X) of the 3D model containing 3.75x10^4^ RAFLS (magenta) and 7.5x10^4^ EC (cyan), including the specific fluorescence signal for each cell type and the output segmentation of the outgrowth area (pink) versus the core area (blue) using trained pixel classifiers in QuPath **(A)**. Ratio of the spheroid outgrowth area to core area *(n=5)* **(B)**. Percentage of the total integrated density of RAFLS **(C)** and ECs **(D)** calculated in the outgrowth area *(n=5)*. Integrated density is the product of mean fluorescence intensity and area. Statistical significance was determined by RM one-way ANOVA (*=*p*<0.05).

Different approaches to quantify spheroid outgrowth were investigated. The **Table 1** summarizes the parameter measurements for each method, also listing their advantages and limitations. Both automated tools “Sprout morphology” plugin and AQuTAS, especially developed for analysis of angiogenesis, compute a unique channel to calculate the number of EC sprouts and total sprout length. While ImageJ plugin provides additional information on branching level, AQuTAS discriminates the number of associated sprouts from the non-associated sprouts. However, these automated methods lack versatility and are not easily adaptable to alternative multicellular systems presenting heterogeneous morphology, including varying cell density, ramification level, amount of connected/detached cells, and core cohesiveness. Further optimization is thus required to adapt these functional automated quantification methods to our model. To standardize spheroid outgrowth in our system, a new quantitative approach discriminating the outgrowth area from the core area by pixel classification was developed (detailed protocol in **Supplementary Material 1**). Outgrowth area was described as the region covered by the cells that migrated out from the core, comprising both associated and non-associated cells. Using trained pixel classifiers, segmentation of the two areas was precisely and robustly performed across all spheroids in a semi-automated way. First, the 3D model of synovial angiogenesis consisting of RAFLS and ECs (32) was replicated and reassessed with the new quantification approach (**Figure 2A**). The spheroids were left unstimulated, stimulated with VEGF/bFGF or cultured in the presence of SF. In response to the addition of VEGF/bFGF, the spheroids displayed enhanced FLS extensions and EC sprouting compared to the unstimulated condition. In the presence of SF, RAFLS and ECs were less dispersed compared to VEGF/bFGF stimulation. However, apparent RAFLS and EC protraction formed a more compact and highly connected network than in the two other conditions (**Figure 2A**). Upon VEGF/bFGF stimulation, a 2-fold increase of the ratio outgrowth to core area was observed compared to basal medium (*p*<0.05) (**Figure 2B**). This increase was also demonstrated in our previous study when assessing the EC cumulative sprout length (32). Following SF stimulation, a 1.5-fold increase in the ratio outgrowth to core area was observed compared to basal medium although this did not reach statistical significance (**Figure 2B**). Additionally, integrated density values of the specifically stained RAFLS and ECs were separately calculated in the outgrowth area. Assuming cell density is proportional to fluorescence intensity, the use of integrated density is indicative of cell density within the measured area. In the presence of VEGF/bFGF, the percentage of integrated cell density of the RAFLS and ECs in the outgrowth area significantly increased by approximatively 15% and 20% respectively compared to the unstimulated condition (*p*<0.05) (**Figures 2C, D**). However, the percentage of integrated density of RAFLS and ECs in the outgrowth area did not vary significantly after addition of SF.

**Table 1:** Selected quantitative image analysis methods to measure spheroid outgrowth, their advantages and limitations.

Tool	Measurement	Advantages	Limitations
**Las X 3D viewer**	Length Counting	Visualisation in 3D True length measured	Requires high-quality Z-stack images No automated method available
**ImageJ « Sprout morphology » plugin** (34)	Nb. of sprouts Total sprout area Total sprout length Average sprout length Branching level	Standardised and automated Information on length and network complexity Reduced experimenter bias	Loss of 3D information Low versatility Requires further optimisation to compute multicellular system
**AQuTAS** (35)	Sprout area Total sprout length Nb. of associated sprouts and non-associated sprouts	Standardised and automated High throughput analysis Information on length and morphology Reduced experimenter bias	Loss of 3D information Low versatility Requires further optimisation to compute multicellular system
**CI Convert/QuPath**	Spheroid outgrowth area Core area Additional features: intensity, cell detection, distances	Semi-standardised and semi-automated Compute multiple channels Robust and versatile	Loss of 3D information No information on length, network complexity or morphology

### 3.2 Incorporation of macrophages results in maintenance of the spheroid structure containing the RAFLS and ECs

**Figure 3:**
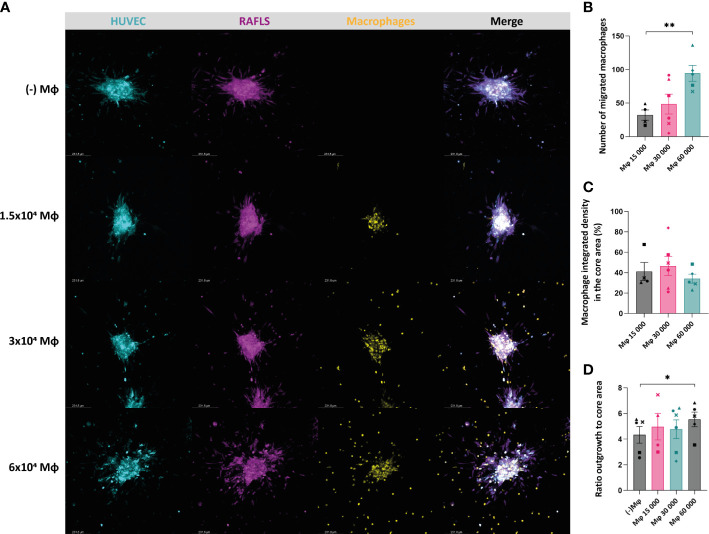
Incorporation of macrophages results in maintenance of the spheroid structure containing the RAFLS and ECs. Spheroids were all cultured in basal medium. Representative confocal Z-stack projection pictures (10X) of the 3D model containing 3.75x10^4^ RAFLS (magenta), 7.5x10^4^ EC (cyan) and macrophages (yellow) **(A)**. Number of macrophages migrating out from the core detected by QuPath software *(n=4-6)* **(B)**. Percentage of the total integrated density of macrophages calculated in the core area *(n=4-6)*. Integrated density is the product of mean fluorescence intensity and area **(C)**. Ratio of the spheroid outgrowth area to core area *(n=4-6)* **(D)**. Statistical significance was determined by RM one-way ANOVA for the analysis of outgrowth ratio and integrated density of RAFLS/EC, and by ratio paired *t*-test for the integrated density of macrophages (*=*p*<0.05, **=*p*<0.01).

In order to recapitulate the *in vivo* RA synovial tissue and the complex interactions of the synovial cells with immune cells, monocyte-derived macrophages were added into the existing 3D model comprising RAFLS and ECs (32). The macrophage ‘M1’-like phenotype was confirmed by determining the expression of CD86 (high levels) and the absence of CD163 expression using flow cytometry (data not shown). Based on the same approach, the cells were co-cultured for 24 hours to allow for spheroid formation. The macrophages merged together with the ECs and RAFLS towards the centre of the well and formed a compact spheroid structure, showing the ability of the model to incorporate macrophages (**Supplementary Material 2**). The formed spheroids containing macrophages were placed in a collagen-based matrix and cultured in basal medium for 40h. Cryosections of the spheroids confirmed the integration of the macrophages and the maintenance of the spheroid structure with the RAFLS and ECs (**Supplementary Material 3**). To investigate the impact of the number of macrophages in the model, a titration of macrophages using the previously established number of RAFLS (3.75x10^4^) and ECs (7.5x10^4^) was performed by adding 1.5x10^4^, 3.0x10^4^, or 6.0x10^4^ macrophages to the co-culture. The spheroid structure incorporating the macrophages was maintained across all ratios, yet a higher number of macrophages outside of the core was also observed when increasing the macrophage input number (**Figure 3A**). The average number of migrated macrophages detected showed a significant increase between 1.5x10^4^ and 6x10^4^ macrophages with a 4-fold difference (*p*<0.01) (**Figure 3B**). Moreover, integrated density values of the specifically stained macrophages were separately calculated in the core area. Assuming cell density is proportional to fluorescence intensity, the use of integrated density is indicative of cell density within the measured area. The percentage of macrophage integrated density in the core area did not vary significantly (**Figure 3C**). This may indicate the limited capacity of the model to contain higher macrophage concentrations using the current spheroid size and cell numbers. To evaluate the effect of macrophage number on spheroid expansion, the ratio outgrowth to core area was calculated. Interestingly, spheroid outgrowth was slightly enhanced when adding 6x10^4^ macrophages (*p*<0.05) (**Figure 3D**). However, the integrated density of RAFLS and ECs in the outgrowth area did not increase significantly (data not shown).

### 3.3 VEGF/bFGF significantly induces spheroid outgrowth in the new 3D model containing macrophages, whereas RASF enhances macrophage containment and compaction

**Figure 4:**
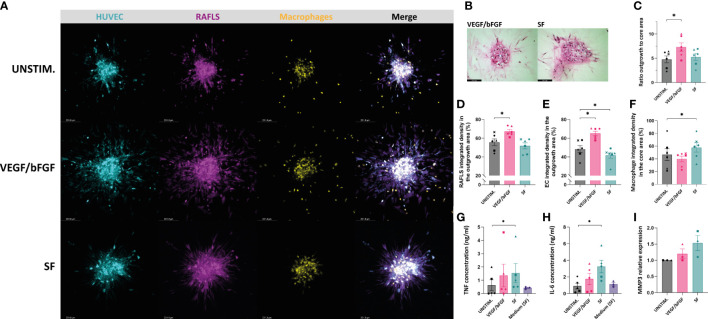
VEGF/bFGF significantly induces spheroid outgrowth in the new 3D model containing macrophages, whereas RASF enhances macrophage containment and compaction. Spheroids were left either unstimulated (UNSTIM.), stimulated with VEGF/bFGF (10 ng/ml) or SF (20%). Representative confocal Z-stack projection pictures of the 3D model containing 3.75x10^4^ RAFLS (magenta), 7.5x10^4^ EC (cyan) and 3x10^4^ macrophages (yellow) **(A)**. Representative pictures of H&E staining in paraffin-embedded spheroid sections for the stimulated condition (5 µm) **(B)**. Ratio of the spheroid outgrowth area to core area *(n=6)* **(C)**. Percentage of the total integrated density of RAFLS **(D)** and ECs **(E)** calculated in the outgrowth area *(n=6).* Percentage of the total integrated density of macrophages calculated in the core area *(n=6)* **(F)**. Concentrations of TNF and IL-6 proteins in the spheroid supernatants as detected by ELISA *(n=5)* **(G, H)**. Relative mRNA expression of MMP3 expressed in fold-change value compared to the unstimulated condition *(n=3)*. The relative expression was normalized to both GAPDH and RPLP0 reference genes using ΔΔCT method (average of the two fold-change values) **(I)**. Statistical significance was determined by RM one-way ANOVA for the analysis of outgrowth ratio and integrated density of RAFLS/EC and by ratio paired *t*-test for the integrated density of macrophages. A Friedman test was applied for TNF and IL-6 protein concentrations (*=*p*<0.05).

After having demonstrated the effects of VEGF/bFGF and SF on spheroid outgrowth in the existing 3D model containing RAFLS and ECs, these stimulation strategies were also tested in the presence of macrophages. After adding VEGF/bFGF, the spheroids containing 3.0x10^4^ macrophages exhibited increased EC sprouting and FLS protraction compared to the unstimulated condition (**Figure 4A**). In the presence of SF, the cells were less spread out and EC sprouts were less pronounced compared to VEGF/bFGF. However, RAFLS extensions formed a dense and highly connected network to a larger extent than the two other conditions whereas the macrophages appeared to be more frequently contained within the spheroid structure (**Figure 4A**). HE staining of spheroid sections revealed cells migrating out from the core upon the two stimulation strategies (**Figure 4B**). Spheroid outgrowth was similarly assessed by measuring the region covered by the cells which migrated away from the core, comprising both associated and non-associated cells. The ratio of the expanded area to the core area was subsequently calculated to describe spheroid outgrowth in medium supplemented with stimuli compared to basal medium. The addition of VEGF/bFGF induced a significant 1.5-fold increase of the spheroid outgrowth ratio compared to the unstimulated condition (*p*<0.05) whereas the addition of SF triggered a lower increase in the spheroid outgrowth ratio than with VEGF/bFGF (**Figure 4C**). Based on this result, the new system with 3.0x10^4^ macrophages is capable of responding to VEGF/bFGF stimulation comparatively to the previous system without macrophages. Of note, the macrophage input numbers 1.5x10^4^ and 6.0x10^4^ did not result in significant changes in the outgrowth ratio following stimulation with VEGF/bFGF or SF (data not reported). Additionally, the percentage of integrated density of the RAFLS and the ECs in the outgrowth area significantly increased by about 10% and 20% respectively upon VEGF/bFGF stimulation (*p*<0.05) (**Figures 4D, E**). Interestingly, in the presence of SF, the EC integrated density in the outgrowth area slightly decreased by approximatively 5% (*p*<0.05) (**Figure 4E**). Moreover, the macrophage integrated density in the core area showed about 15% increase upon SF stimulation compared to the unstimulated condition (*p*<0.05) (**Figure 4F**). These changes in cell migration indicate greater cell compaction and containment of the ECs and the macrophages within the spheroid structure when the cells are in contact with SF. Furthermore, the average protein concentrations of TNF and IL-6 measured in the supernatant of the spheroid cultures revealed a 2-fold and 3-fold increase respectively following SF stimulation compared to the unstimulated condition (p<0.05) and similarly to the medium containing 20% SF solely. An increase in mRNA relative expression of matrix metalloproteinase-3 (MMP3) was determined upon stimulation, with up to 1.5-fold change in the presence of SF compared to the unstimulated condition.

## 4 Discussion

In the current study, we described a novel spheroid-based 3D model of RA synovial tissue incorporating RAFLS, ECs and monocyte-derived macrophages, which could be used as a tool for preclinical studies and translational research in RA, and potentially also other inflammatory joint diseases. We also describe a new semi-automated and standardised quantification method by machine learning to evaluate spheroid outgrowth in the 3D model. This quantitative analysis measured the area covered by the cells migrating out from the core, including RAFLS and ECs as they are known to coordinate during cell migration in a collagen matrix (32, 37). The ratio of the expanded area to core area and the percentage of cell-specific integrated densities in the different regions were calculated and used to analyze the response of the new model to different stimuli. Interestingly, RAFLS and ECs exhibited an activated migratory phenotype under unstimulated conditions. In line with our previous study (32), this was expected as RAFLS intrinsically harbor an invasive phenotype, as well as producing pro-angiogenic factors such as VEGF/bFGF, and pro-inflammatory cytokines exerting synergic effect with TNF in producing VEGF (14, 17, 18, 20). The addition of VEGF/bFGF further increased both the spheroid outgrowth ratio and the percentage of integrated density of the RAFLS and ECs in the outgrowth area compared to the unstimulated condition. Stimulation with SF had a less pronounced effect on these measurement parameters. The area and intensity measurements alone may not provide enough information to fully describe differences in spheroid outgrowth morphology in the presence of SF. For example, sprout number, sprout length, branching level and distinction between connected and detached cells migrating out from the core could provide additional insights to investigate the effect of SF stimulation in our system. The upregulation of pro-inflammatory cytokines and chemokines indicating RA synovial inflammation could also be evaluated in the presence of SF compared to the unstimulated condition. The expression of adhesion molecules including different integrins and cadherins, together with the production of matrix metalloproteinases (MMPs) and reactive oxygen species (ROS) may also be indicative of the inflammatory response to SF.

The introduction of macrophages into the existing model demonstrated tight interaction with the ECs and RAFLS within the spheroid structure. When increasing the macrophage input number, higher numbers of macrophages were detected outside of the core while the specific integrated intensity in the core seemed to be reduced. This suggests the limited capacity of the model to integrate macrophages at high concentrations. If necessary, the input number of RAFLS and ECs may need to be increased for a more optimal integration of the macrophages. Furthermore, in the case of a high macrophage concentration (i.e. higher than the number of RAFLS), spheroid outgrowth was slightly promoted compared to the absence of macrophages. In the development of a model of RA synovial tissue, a low number of macrophages may be preferred to establish a baseline in which cell interactions driving synovial inflammation are controlled under unstimulated conditions. However, in studies investigating angiogenesis, a high number of macrophages activating EC sprouting may be desirable. Thus, it is important to note that the optimal cell ratio may differ depending on the specific research question or application. These results confirm the important role of macrophages in RA synovial inflammation and the necessity of integrating these immune cells in a 3D model of RA synovial tissue. In the *in vivo* situation, macrophages are either tissue-resident or derived from circulating blood monocytes infiltrating the inflamed tissue. The latter are widely recognized to interact with ECs and RAFLS present in the inflamed synovium *via* the production of a plethora of cytokines inducing cell activation, proliferation and angiogenesis (14, 40–42). In particular, TNF can induce the proliferation of FLS *via* the nuclear factor kappa-B (NF-κB) signaling pathway, whereas transforming growth factor (TGF)-β promotes FLS migration and invasion (2). In contrast, tissue-resident macrophages typically lack the pro-inflammatory activation profile and are characterized by an anti-inflammatory phenotype (14, 40–42). Nonetheless, diversity of the macrophage compartment in RA synovium extends beyond the initially described so-called ‘M1’ (pro-inflammatory) and ‘M2’ (anti-inflammatory) macrophages. Recent single-cell transcriptome sequencing analyses have shown the striking heterogeneity of synovial tissue macrophage subsets (43). Different macrophage subsets can be thus integrated in this new 3D model to investigate their specific contributions to synovial inflammation and angiogenesis in close interaction with ECs and RAFLS. In the current study, the macrophages were differentiated towards a pro-inflammatory phenotype in the presence of GM-CSF to further simulate synovial inflammation. From an opposite perspective, it would be interesting to investigate the effect of the anti-inflammatory counterpart (i.e. cultured in M-CSF) or further polarized into different subsets (44, 45). Like monocyte-derived macrophages, trafficking lymphocytes strongly contribute to the excessive production of pro-inflammatory factors in the inflamed tissue and are responsible for disease progression (13). Therefore, other types of immune cells, such as B and T cells, could be similarly incorporated into the model to further recapitulate the heterogeneity of immune cell infiltrates in the RA synovial tissue and study the interaction between these cell types.

Stimulation of the new 3D model incorporating macrophages with SF induced an increase in both TNF and IL-6 protein concentrations in the supernatant as well as MMP3 transcription in the spheroids compared to basal medium. The upregulation of pro-inflammatory cytokines and MMP3 upon SF stimulation demonstrates the capacity of the SF cocktail to induce an inflammatory response and matrix degradation potential in the spheroids. The presence of SF also triggered an interesting change in cell migratory phenotype. The RAFLS extensions formed a dense network closely attached to the core whereas the EC sprouts were slightly reduced and macrophages were more contained within the core compared to the unstimulated condition. The tight interaction between the cell types forming a more compact spheroid structure may be due to the upregulation of adhesion molecules induced by the presence of pro-inflammatory mediators in the SF. Kiener et al. have previously described the cocompaction of FLS and macrophages into the micromass lining layer following TNF exposure (28) found at high levels in SF (46). RAFLS and macrophages closely associate and co-inhabit in the synovial tissue microenvironment where RAFLS secrete GM-CSF, IL-6, IL-8, CXCL10 and CCL2 that can attract macrophages (14). Moreover, the macrophage migration inhibitory factor (MIF) is found at elevated levels in SF and is known to inhibit the random migration of macrophages, as well as promoting the proliferation and survival of synovial fibroblasts (25, 47, 48). Further investigation is required to dissect the cell interactions underlying cell migration and arrangement when the spheroids are stimulated with SF. Of note, alternative simulation strategies can be implemented to asses both on-target and off-target effects of drug candidates in the model. For example, exposure to TNF is often chosen to mimic the inflammatory conditions in RA, yet other pivotal cytokines like IL-6 and IL-17 could be equivalently used to induce synovial inflammation.

Ultimately, this new 3D model of RA synovial tissue may be useful to study specific signaling pathways involved in RA pathogenesis and bring novel insights for the development of new therapeutic targets. Many cytokines secreted by monocyte-derived macrophages and RAFLS are used in practice as targets for RA treatment such as TNF, IL-6 and IL-1 inhibitors (2). A large panel of promising molecular targets implicated in the interaction between macrophages, RAFLS and ECs is currently being actively explored for drug discovery, including interleukins, chemokines, and other proteins such as kinases and glycoproteins, besides small molecular metabolites (2). Therefore, extensive preclinical studies are required for proving the therapeutic or diagnostic potential of these targets before performing clinical trials. This fully human 3D multicellular system may be a promising alternative to animal models of RA which present limitations when it comes to testing biologics highly specific for human target proteins (25, 49). It offers an opportunity for early-stage implementation of drug testing in preclinical development, thereby reducing the need for animal experiments. This comprises the assessment of specific inhibitors, including small molecule inhibitors and biologics, as well as testing the binding of molecular imaging compounds to quantify predictive diagnostic and theranostic biomarkers in RA.

## Data availability statement

The original contributions presented in the study are included in the article/**Supplementary Material**. Further inquiries can be directed to the corresponding author.

## Author contributions

All authors met the criteria for authorship set by the International Committee of Medical Journal Editors. Conceived and performed the methodology and experiments: EP and LR. Helped in performing the experiments: FK. Analyzed the data: EP. Wrote the original draft: EP. Writing, review and editing: EP, JanV, ST, JacV. All authors revised the manuscript for important intellectual content and approved the submitted version.
