# Supplementary material for: A novel 3D spheroid model of rheumatoid arthritis synovial tissue incorporating fibroblasts, endothelial cells, and macrophages
Source: Front Immunol. 2023 Jul 20;14:1188835. doi: 10.3389/fimmu.2023.1188835 (PMC10402919; doi:10.3389/fimmu.2023.1188835)

Supplementary Material

A novel 3D spheroid model of rheumatoid arthritis synovial tissue incorporating fibroblasts, endothelial cells, and macrophages

E.M.L Philippon[1,2], L.J.E van Rooijen[1,2], F. Khodadust[1], J.P. van Hamburg[1,2], C.J van der Laken[1] and S. W. Tas[1,2*]

[1]Amsterdam UMC, Department of Rheumatology & Clinical Immunology, Amsterdam Rheumatology & immunology Center, Netherlands

[2]Amsterdam UMC, Department of Experimental Immunology, Netherlands

*** Correspondence:**

Corresponding Author
s.w.tas@amsterdamumc.nl

Supplementary Material 1: Detailed protocol of the quantitative image analysis using machine learning (QuPath) to quantify spheroid outgrowth and morphological changes.

1/ Converting confocal images into IJ Hyperstack TIFF format

1. Open CI Convert software (https://software.cellularimaging.nl/ci-convert/)
2. In the "Convert LIF/XLEF to SVS/TIFF/ImageJTIFF" window, import your LAS-X File (LIF or XLEF)
3. Select the Output File Type: IJ Hyperstack TIFF (8/16bits)
4. Choose the most suitable Z-Stacks Projection (Extended Depth of Focus, EDF (BF/PH)/Maximum, EDF (Gray)/Maximum…)
5. Click on Preview Image to check the output picture
6. Select the images to convert and click on Convert Selected Images

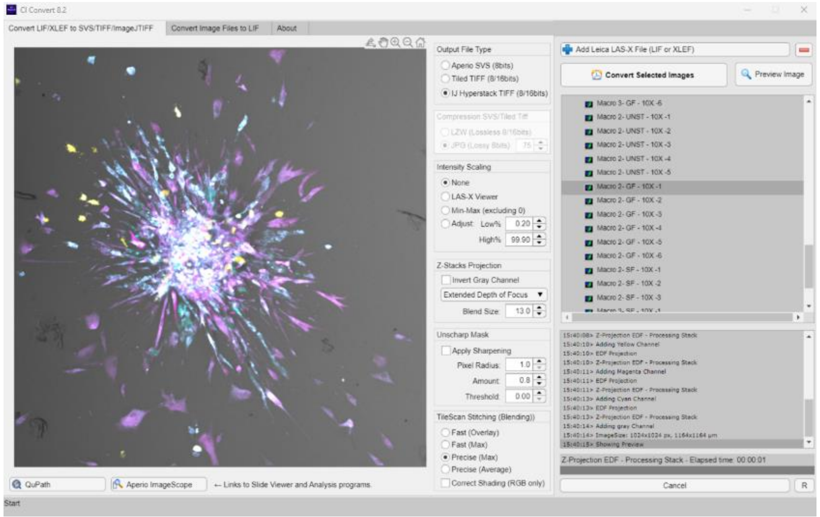

2/ Segmentation of the spheroid outgrowth and core areas by pixel classification

7. Open QuPath software
8. Create a new project and drag the CI convert pictures to the QuPath window.
9. Set the image type as “Fluorescence” to preserve the individual fluorescence channels alongside with the bright-field channel
10. To train the pixel classifier, select a pool of 3-6 images that capture the diversity of the spheroids in terms of cell density, morphology, signal intensity, and that also includes artefacts for the pixel classifier to learn how to discriminate.
11. Classify > Training image > Create training image > “Unclassified”
12. Duplicate the training image (one training by region of interest)
13. Go to Annotations and create the classes: “outgrowth” and “core”
14. Using the brush, create 2-3 annotations corresponding to the suitable region of interest
15. Create 2-3 annotations corresponding to the background “Ignore*”

Annotations to train the pixel classifier for the “Outgrowth” area

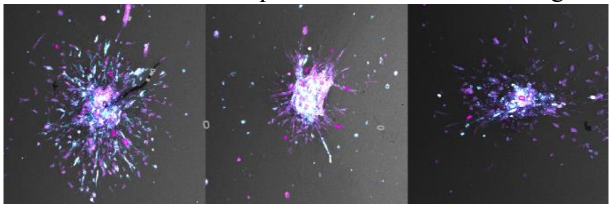

Annotations to train the pixel classifier for the “Core” area

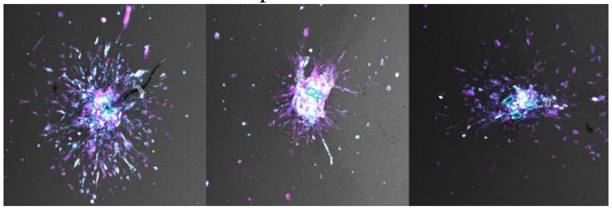

16. Classify > Pixel classification > Train pixel classifier (a small library of classifiers may be needed to analyse a high diversity in spheroid morphology)
17. Define the parameters of the Pixel Classifier (these are exhaustive and may be modified according to the experiment)

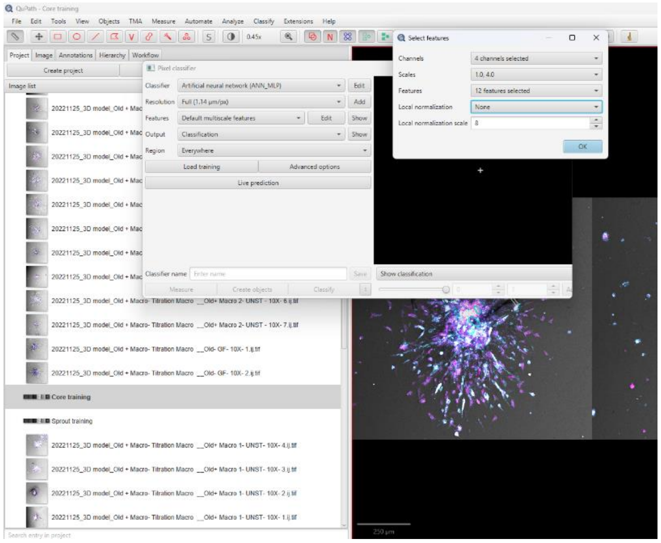

Guideline :

- Classifier : Random Trees or Artificial Neural Network
- Resolution: Full (outgrowth), Very High/High (core)
- Features: 4 channels, scales (1.0,4.0), 12 features selected

18. Click on Live Prediction

19. Create additional annotations to teach the pixel classifier to recognize the region of interest (the chart pie should remain evenly distributed between the region and the background) until the segmentation is accurate enough
20. Name and save the trained pixel classifier

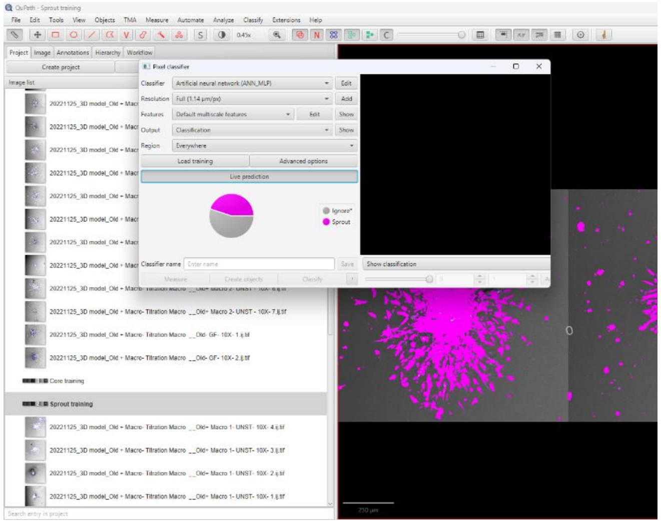

21. Go back to the images and make sure the analysis will be applied to a single spheroid. If another spheroid is visible, create a closed polygon annotation to isolate the spheroid of interest.
22. Select an image to analyse
23. Classify > Pixel classification > Load pixel classifier
24. Choose the “Core” classifier and look for the most accurate classifier
25. Create Objects > All annotations
26. Define the minimum object size to exclude smaller non-related objects and minimum hole size to fill in the holes if needed
27. Tick the box “Set new objects to selected” (the new annotation “Core” will appear in the “Annotations” window and will be automatically selected)
28. Object > Annotations > Make inverse (create a region annotation of everything except from the “Core”)
29. Repeat steps 23-26 with the “Outgrowth” classifier
30. Check whether the segmentation is accurate by looking at the superposition of the two annotations to the original picture
31. To apply the trained pixel classifiers on the whole dataset, go to Automate > Show script editor
32. Write the following script:

```
setImageType('FLUORESCENCE')
selectAnnotations() #in case it needs to consider the closed polygon annotation isolating the spheroid
createAnnotationsFromPixelClassifier("Core classifier", X, Y, "SELECT_NEW") #define X, Y parameters
makeInverseAnnotation() #create region annotation of everything except from the “Core”
createAnnotationsFromPixelClassifier("Outgrowth classifier", X, X) #define X, Y parameters
```

33. Click on Run > Run for project, and select the project images
34. Measure > Export measurements
35. Select the analysed images, choose the output file and the export type “Annotations”
36. Use the option “Populate” to only include the parameters of interest (Image, Class, Area,...)

3/ Additional measurement features

37. Use the plug-in Analyse > Calculate features > Add intensity features to calculate the mean fluorescence intensity for each channel of the “Outgrowth” and the “Core” annotations
38. Select the fluorescence channels, the basic features > Run
39. To apply the plug-in on the whole dataset, go to Automate > Show workflow command history (the command *runPlugin('qupath.lib.algorithms.IntensityFeaturesPlugin',……)* with the set parameters will appear)
40. Click on Run > Run for project, and select the project images (the command can be also integrated in the previous script to be directly applied after segmentation)

41. Use the plug-in Analyse > Cell detection > Cell detection to detect macrophages in the “Outgrowth” annotation

42. Define the macrophage fluorescence channel, nucleus, intensity and cell parameters > Run (detection objects corresponding to the cells detected will be created and counted)
43. To apply the plug-in on the whole dataset, go to Automate > Show workflow command history (the command *runPlugin('qupath.imagej.detect.cells.WatershedCellDetection'…….)* with the set parameters will appear)
44. Integrate the command in the following script:

```
setImageType('FLUORESCENCE');
selectAnnotations(); #in case it needs to consider the closed polygon annotation isolating the spheroid
createAnnotationsFromPixelClassifier("Core Classifier", X, X, "SELECT_NEW") #define X, Y parameters
runPlugin('qupath.lib.plugins.objects.DilateAnnotationPlugin',
'{"radiusMicrons":20.0,"lineCap":"ROUND","removeInterior":false,"constrainToParent":false}')
#extend the “Core” of 20 microns to exclude the macrophages at the border
#the new annotation created will be called “Core”
#the selection is automatically maintained on the previous “Core” annotation
classifySelected('Core strict') #change the name of the previous “Core” annotation
selectObjectsByClassification("Core") #select the new “Core” annotation
resolveHierarchy() # make sure the new “Core” annotation is included within the closed polygon annotation
selectObjectsByClassification("Core") #select the new “Core” annotation
makeInverseAnnotation() #create region annotation of everything except from the “Core”
runPlugin('qupath.imagej.detect.cells.WatershedCellDetection'…….) #command of the workflow history to detect macrophages
detectionToAnnotationDistances(true) #calculate the distance of the macrophages to the core
```

45. Click on Run > Run for project, and select the project images
46. Measure > Export measurements
47. Select the analysed images, choose the output file and the export type “Annotations” (or “Detections” to export the data related to the macrophages such as the distance to the “Core”)
48. Use the option “Populate” to only include the parameters of interest (Image, Class, Intensity, number of detected cells,...)

Supplementary Material 2: Representative pictures of spheroid formation at different time points containing RAFLS and ECs (A) with the incorporation of macrophages (B). $3.75x10^4$ RAFLS (no colour), $7.5x10^4$ ECs (red) and $3x10^4$ macrophages (green) were distributed into a 96-well plate in Methocell solution and incubated in the Incucyte® Live-Cell Analysis System for 24 hours where successive pictures were taken every 30 min. Both conditions showed rapid migration of the dispersed cells towards the center of the well to form a loose aggregate. However, the macrophages appeared to be slower to migrate than the ECs. After 3h of incubation, most evaof the ECs had merged together whereas an important proportion of macrophages were still spread out. After 24h, most of the spheroids had undergone compaction and an outer layer of cells was visible. Overall, the addition of macrophages did not seem to affect the speed of spheroid formation.

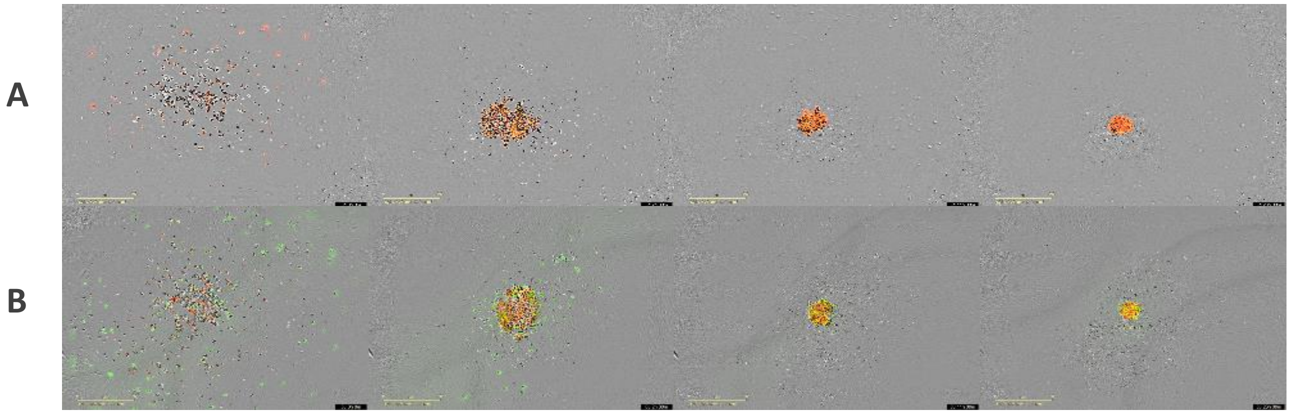

Supplementary Material 3: Cryosections of spheroids showing maintenance of the macrophages within the spheroid structure with the RAFLS and ECs. The formed spheroids containing $3.75x10^4$ RAFLS (magenta), $7.5x10^4$ ECs (cyan) and $3x10^4$ macrophages (yellow) were placed in a collagen-based matrix using the sandwich method and cultured in basal medium for 40h. The cells were incubated with specific CellTracker™ dyes (Invitrogen) prior spheroid formation to allow for subsequent visualization by fluorescence imaging. The collagen gels containing the spheroids were fixed with 4% PFA and embedded in Tissue-Tek O.C.T. Compound for snap-freezing. Cryosections (5 µm) were made using a cryostat and imaged with the Leica TCS SP8-X confocal microscope (20X).

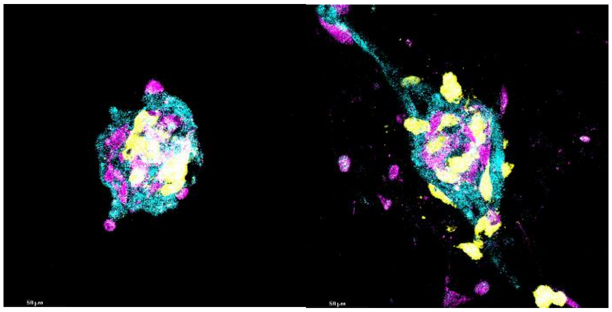

Supplement: Supplementary file 1 [file DataSheet_1.pdf]
